# Natural Compounds Interacting with Nicotinic Acetylcholine Receptors: From Low-Molecular Weight Ones to Peptides and Proteins

**DOI:** 10.3390/toxins7051683

**Published:** 2015-05-14

**Authors:** Denis Kudryavtsev, Irina Shelukhina, Catherine Vulfius, Tatyana Makarieva, Valentin Stonik, Maxim Zhmak, Igor Ivanov, Igor Kasheverov, Yuri Utkin, Victor Tsetlin

**Affiliations:** 1 Shemyakin-Ovchinnikov Institute of Bioorganic Chemistry, Russian Academy of Sciences, Moscow 117997, Russia; E-Mails: kudryavtsev@ibch.ru (D.K.); ner-neri@mail.ru (I.S.); chai.mail0@gmail.com (I.I.); iekash@mx.ibch.ru (I.K.); utkin@mx.ibch.ru (Y.U.); 2 Institute of Cell Biophysics, Russian Academy of Sciences, Pushchino 142290, Russia; E-Mail: vulfius@gmail.com; 3 Elyakov Pacific Institute of Bioorganic Chemistry, Far East Branch of the Russian Academy of Sciences, Vladivostok 690022, Russia; E-Mails: makarieva@piboc.dvo.ru (T.M.); stonik@piboc.dvo.ru (V.S.); 4 SME Syneuro, Moscow 117997, Russia; E-Mail: mzhmak@gmail.com

**Keywords:** α-conotoxins, low-molecular weight agonists and antagonists, α-neurotoxins, nicotinic acetylcholine receptors, snake venom phospholipases A2, three-finger Ly6 proteins

## Abstract

Nicotinic acetylcholine receptors (nAChRs) fulfill a variety of functions making identification and analysis of nAChR subtypes a challenging task. Traditional instruments for nAChR research are d-tubocurarine, snake venom protein α-bungarotoxin (α-Bgt), and α-conotoxins, neurotoxic peptides from *Conus* snails. Various new compounds of different structural classes also interacting with nAChRs have been recently identified. Among the low-molecular weight compounds are alkaloids pibocin, varacin and makaluvamines C and G. 6-Bromohypaphorine from the mollusk *Hermissenda crassicornis* does not bind to *Torpedo* nAChR but behaves as an agonist on human α7 nAChR. To get more selective α-conotoxins, computer modeling of their complexes with acetylcholine-binding proteins and distinct nAChRs was used. Several novel three-finger neurotoxins targeting nAChRs were described and α-Bgt inhibition of GABA-A receptors was discovered. Information on the mechanisms of nAChR interactions with the three-finger proteins of the Ly6 family was found. Snake venom phospholipases A_2_ were recently found to inhibit different nAChR subtypes. Blocking of nAChRs in *Lymnaea stagnalis* neurons was shown for venom C-type lectin-like proteins, appearing to be the largest molecules capable to interact with the receptor. A huge nAChR molecule sensible to conformational rearrangements accommodates diverse binding sites recognizable by structurally very different compounds.

## 1. Introduction

Nicotinic acetylcholine receptors (nAChRs) have been in the focus of researchers interested in fundamental problems of neurobiology, pharmacology and drug design for several decades. The reason is a widespread distribution of different types and subtypes of muscle-type, neuronal and so-called “non-neuronal” nAChRs in the organism and their involvement in such diverse normal physiological processes as muscle contraction, cognitive functions such as learning and memory, participation in the pain signal transduction, neuroprotection and regulation of the immune responses (see recent reviews [1,2,3]). Malfunctioning of nAChRs due to impaired expression levels, mutations or interactions with “wrong” partners (e.g., β-amyloid) are more or less directly associated with diseases such as myasthenia gravis, epilepsy, schizophrenia, Alzheimer’s and Parkinson diseases and nicotine addiction [4,5,6]. Spatial structure (cryo-electron microscopy at a resolution of 4 Å) is available only for the muscle-type nAChR from the electric organ of Torpedo marmorata ray [7,8]. Nevertheless, very rich information is compiled about the three-dimensional organization, topography of binding sites and mechanisms of action for the nAChR major types based on the Torpedo receptor 3D structure, computer modeling and the X-ray structures of the acetylcholine-binding proteins (AChBPs) [9,10], which are excellent models of the ligand-binding domains of all nAChRs. Even more interesting are the AChBP crystalline complexes with various cholinergic agonists: carbamylcholine and nicotine [11], epibatidine [12,13], as well as with antagonists: d-tubocurarine [14], snake venom α-neurotoxins [15,16], and α-conotoxins from Conus marine snails [10,17,18]. These studies have provided valuable information about the topography of the nAChR ligand-binding sites and the interacting surfaces of the bound ligands, thus shedding light on the mechanisms of action and giving hints for the design of novel drugs. It is appropriate to mention here that both low-molecular compounds and α-neurotoxins were instrumental at the earlier stages of isolation and characterization of nAChRs (see reviews [19,20]) and their role of accurate pharmacological tools are retained at the present time as well. For example, fluorescent derivatives of α-bungarotoxin are excellent tools for detecting neuronal α7 nAChRs [21], cytisine allows to distinguish between certain heteromeric neuronal nAChRs [22]. The most excellent tools for identification and quantification of a variety of neuronal nAChRs (even those differing in the stoichiometry of the same subunits) are naturally-occurring α-conotoxins and their synthetic analogs [23,24,25]. The purpose of this mini-review is to briefly consider new results on low molecular compounds from natural sources as well as on peptide and protein neurotoxins, focusing on our recent results in this field.

## 2. Low-Molecular Weight Agonists and Antagonists of nAChRs

### 2.1. Short Summary of Well-Known Agonists and Antagonists of nAChRs

We would like to start this section with a summary of those compounds, which are well known and continue to play important roles in research on the nAChRs (Table 1). 

**Table 1:** Naturally occurring agonists and antagonists of different nicotinic acetylcholine receptors (nAChRs).

Compound	Source	Activity
acetylcholine	-	Prototypic agonist at all nicotinic receptors
choline	-	Agonist at α7 and muscle nAChRs
nicotine	*Tobacco* plant	Agonist at most nAChR subtypes; antagonist at α9 nAChR
epibatidine	*Epipedobates* frogs	Agonist at most nAChR subtypes; antagonist at α9 nAChR
cytisine	Plants of *Fabaceae* family	Partial agonist at neuronal nAChRs
anatoxin-a	Cyanobacteria	Non-selective agonist of nAChRs
anabaseine	Certain species of ants and marine worms	Agonist at neuronal nAChRs
d-tubocurarine	*Chondodendron tomentosum* plant	Non-selective antagonist
coniine	*Conium maculatum* plant	Antagonist at muscle nAChRs
pinnatoxins, 1,3 desmethyl spirolide, gymnodimines	dinoflagellates	Non-selective antagonists
pictamine	*Clavelina picta* ascidian	Antagonist at neuronal nAChRs

### 2.2. nAChR Antagonists and Agonists of Marine Origin

Of special interest are compounds from various marine sources. For many of them the action on nAChRs has been discovered relatively recently. Groups of compounds such as pinnatoxines, spirolides and gymnodimines produced by dinoflagellates could easily be accumulated by pabular shellfish and cause serious poisoning [26]. Their toxicity appears to be realized through the interaction with nAChRs [27] and muscarinic acetylcholine receptors (mAChRs) [28] characterized by nanomolar affinity, whereas cytotoxic effects were absent [29].

**Figure 1:**
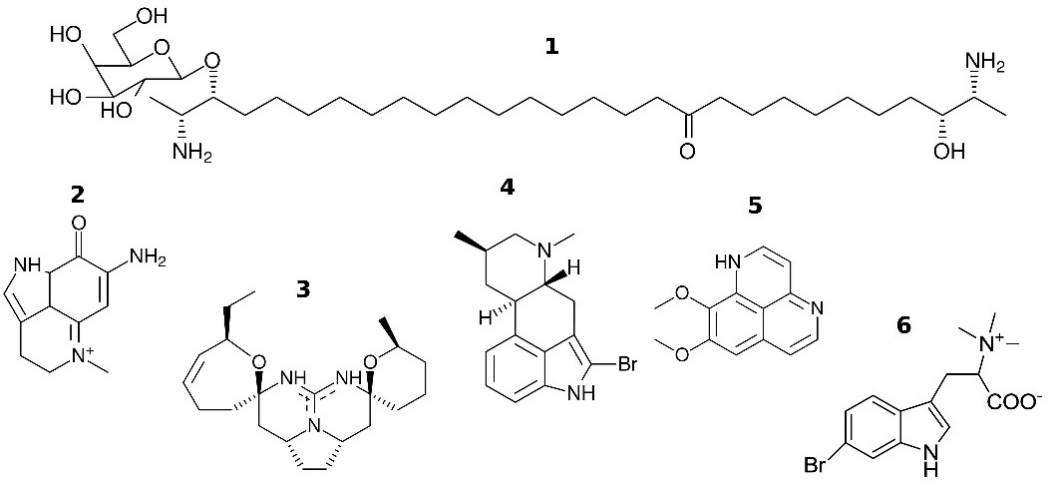
Chemical structures of cholinergic ligands from marine sources: **1**—rhizochalin; **2**—makaluvamine C; **3**—crambescidin 359; **4**—pibocin; **5**—aaptamine; **6**—6-bromohypaphorine.

In the recent work [30] it has been shown that several marine natural compounds (some are shown in Figure 1) potently inhibit muscle-type and α7 nAChRs. Among them are sphyngolipid rhizochalin (**1**) and its aglycone, pyrroliminoquinones makaluvamines C (**2**) and G, cyclic guanidines crambescidin 359 (**3**) and monanchocidin, ergoline derivative pibocin (**4**) and naphthyridine derivative aaptamine (**5**). Therefore, nAChRs may be important targets for marine toxins. In view of the great number and versatility of natural products, the question how to predict whether one or another compound will interact with nAChRs is becoming crucial in optimizing a search for potential drug candidates. Molecular docking of low-molecular weight compounds to the X-ray structures of AChBP provides reasonable predictions of the affinities at a low computational cost [30,31]. Such computer modeling was the first step in the analysis of the above-mentioned compounds.

All compounds, for which we revealed interactions with the muscle-type and α7 nAChRs, acted as antagonists. Finding agonists, especially selective ones, might be even more interesting, because activation of neuronal nAChRs at certain conditions can be considered as a way of treating neurological pathologies [32]. For example, α7 nAChR activator EVP-6124 shows positive results in clinical trials of treating conditions such as schizophrenia [33]; ABT-594 is a less toxic epibatidine analog with analgesic properties [34]. Recently we found that 6-bromohypaphorine (6) from marine nudibranch *Hermissenda carassicornis* acts as an agonist at human α7 nAChR and, moreover, possesses a certain selectivity towards distinct nAChR subtypes [35].

## 3. α-Conotoxins and Other Peptides Interacting with nAChRs

### 3.1. Naturally-Occurring α-Conotoxins from Conus Marine Snails

Among the peptides capable of interacting with one or another nAChR subtype are apolipoprotein E (ApoE) fragments, calcitonin gene related peptide (CGRP), β-amyloid peptides 1–40 and 1–42 and some others, which were presented in detail in the review [24]. However, peptide neurotoxins (conotoxins and conopeptides) from venomous *Conus* marine snails are the most important and widely applied. Various types of α-conotoxins block nAChRs, and their advantage over all other cholinergic compounds is that some representatives of this family not only allow to distinguish muscle-type nAChRs from various neuronal subtypes, but also show more or less strict selectivity towards a distinct neuronal subtype. The importance of α-conotoxins both for fundamental research and for medicinal applications was discussed in several reviews [36,37,38]. In particular, α-conotoxin RgIA, which binds to the α9 nAChRs, is investigated as a potential analgesic [39]. However, naturally occurring α-conotoxins show a combination of high affinity with strict selectivity to a particular nAChR subtype, which is extremely rare. For this purpose several solutions have been proposed. In recent years, the number of available α-conotoxin sequences has increased considerably due to getting the *Conus* species from new areas (for example, from coasts of South Africa, South China, Australia, India, Vietnam, and Mexico) and isolating novel peptides either from minute amounts of venoms or by the analysis of mRNA and deduction of the putative conotoxins from the sequences of the precursors [40,41,42,43,44,45,46,47,48].

### 3.2. Design and Synthesis of More Potent and Selective α-Conotoxin Analogs

Another approach to increase the affinity and/or selectivity of α-conotoxin towards a desired nAChR subtype consists of introducing one or several substitutions into the amino-acid sequences of the naturally occurring α-conotoxins. One of the first analogs with the increased selectivity for the α7 nAChR, namely ArIB[L11, D16], was obtained by introducing two substitutions into naturally-occurring α-conotoxin ArIB [49]. The affinity of α-conotoxin ImI for α7 nAChR was increased by about 10-fold by analyzing a combinatorial library [50]. Similarly, in a combinatorial library, a potent and selective analog inhibiting α3β4 nAChR was identified [51]. Introduction of three substitutions to α-conotoxin MII acting on the α3β2 and α6 subunit-containing nAChRs made it much more selective towards the latter subtypes [52]. Moreover, an α-conotoxin analog has recently been designed which not only distinguishes the α6 subunits but is also more selective to α6β2 subtypes than α6β4 ones [53].

It should be noted that for detecting the involvement of a particular nAChR subtype in the physiological process it is sufficient to apply a selective α-conotoxin, which would block the current induced in the receptor of interest. On the other hand, localizing that receptor in brain and other tissues requires an appropriate label (radioactive or fluorescent) to be attached to α-conotoxin and to preserve a sufficiently stable binding of the modified α-conotoxin to the examined tissue. This requirement creates additional problems because most of α-conotoxins, even those showing a high affinity, are characterized by very fast rates of association and dissociation from their nAChR targets. In several cases this problem was successfully solved by introducing suitable labels into the sequences of naturally occurring α-conotoxins. For instance, radioactive analogs of a relatively slow dissociating α-conotoxin MII are widely used for mapping α3 and α6 subunits-containing nAChRs in tissue slices [54,55]. However, fluorescent labeling modifies α-conotoxins and restricts their area of application mostly to cytochemistry. For example, Alexa Fluor 546-BuIA was applied to stain β4 subunit-containing nAChRs on human chromaffin cells of the adrenal gland [56], FITC-MII was used to study nAChRs in the neuroblastoma cell line SH-SY5Y [57] and Cy3- and Alexa Fluor 546-ArIB[L11, A16] labeled α7 nAChRs in cultured hippocampal neurons [58]. 

In most cases a search for suitable α-conotoxin analogs proceeds along the “trial and error” roads, and the success is hardly predictable. Because the first crystal structure of α-conotoxin in complex with AChBP was that of α-conotoxin PnIA[L10, K14] [10], we continued a search for more efficient α-conotoxin analogs based on the X-ray data and applied various methods of computer modeling. Using earlier available computational methods, we managed to increase the affinity of PnIA[R5,L10,R14] both for the AChBP and α7 nAChR [59] (see also Table 2). An important step was the comparison of affinities for α-conotoxin analogs assessed either via competition with radio-iodinated α-Bgt or with an iodinated form of a new PnIA analog. In the latter case the measured affinities were about 10-fold higher (Table 2). This is of practical interest because in most cases the affinities of novel compounds for AChBPs or α7 nAChR are measured via their competition with radio-iodinated α-Bgt. However, because of the virtually irreversible attachment of the latter to the above targets, measurements are difficult and should be made very quickly. A novel program for assessing the protein-protein interactions [60] named “protein surface topography” was created at the Shemyakin-Ovchinnikov Institute. The essence of this technique is an approximate representation of the structures of small peptides as a sphere and the physico-chemical properties (charges and hydrophobicity) over their surface as projection maps. A comparative analysis of bioactive peptide groups with the help of these maps makes it possible to detect the fine specific structure-function features in a set of molecules similar in their structures and properties, as well as to find out their correlations with peptide activities towards certain targets. We applied the program in an attempt to create novel PnIA analogs of higher affinity for α7 nAChR and/or AChBPs. The data on activities (as IC_50_ values) of 16 α-conotoxin PnIA analogs from [59], as well as the available published affinity parameters for the α-conotoxins ArIA, ArIB, GID, TxIA and several others (39 molecules in total) were used in computations (for a detailed description of “protein surface topography” application towards α-conotoxin PnIA refer to [61]). The constructed “averaged” map suggested that the introduction of positively charged residues in positions 5, 9 and 14 of α-conotoxin PnIA would “optimize” the electrostatic properties and result in a gain of affinity towards α7 nAChR and AChBPs. Apart from these substitutions, it is known that the [A10L] mutation improves the target affinity. In total, three new analogs—PnIA[R9], PnIA[R9, L10] and PnIA[R5, R9, L10, R14]—were selected (Table 2).

**Table 2:** Activity of α-conotoxin PnIA analogs tested in competitive radioligand or electrophysiology assays. The selected peptides were evaluated regarding their ability to compete with [^125^I]-labeled α-bungarotoxin ([^125^I]-αBgt) or radioiodinated α-conotoxins for binding to AChBPs and human α7 nAChR. In electrophysiological experiments, the capability of the peptides to decrease the nicotine-induced current was estimated. The presented IC_50_ values (in nM) were calculated using ORIGIN 7.5 with the joint data from two or three independent experiments for each analog. The color marks the residues for substitutions.

Substitutions in PnIA	Affinity (IC_50_, nM) for				
	AChBPs in Competition with [^125^I]-αBgt		Human α7 nAChR		
	L. stagnalis	A. californica	in competition with		in electrophysiology
			[^125^I]-αBgt	[^125^I]-α-conotoxin	
	*α-conotoxin PnIA*: GCCSHPPCAANNPDYC-NH_2_				
[H5]	220	3.1	26,000	-	-
[H5, R14]	2,900	1,400	21,000	-	-
[L10]	200	55	14,000	-	-
[D5, L10]	35,000	5,200	>100,000	-	-
[R5, L10]	180	305	12,000	-	-
[D7, L10]	>>100,000	63,000	>>100,000	-	-
[R7, L10]	160,000	1,250	>>100,000	-	-
[L10, K14]	8.2	47	7,200	1,800 ^1^	260
[D5, R7, L10]	38,000	51	275,000	-	-
[D5, R7, V10]	6,400	45	>100,000	-	-
[R5, D7, L10]	56,000	28,000	>>100,000	-	-
[R5, L10, R14]	430	1,400	670	60 ^1^	-
[D5, R7, L10, R14]	1,200	46	23,000	-	-
[R5, D7, L10, R14]	4,100	3,200	72,000	-	-
[R9]	58	41	2,400	1,490 ^2^	27
[R9, L10]	18	47	270	36 ^2^	17
[R5, R9, L10, R14]	1.2	24	860	390 ^2^	27

^1^ in competition with [^125^I]-α-conotoxin PnIA[R5, L10, R14]; ^2^ in competition with [^125^I]-α-conotoxin PnIA[R9, L10].

The most active ligand for AChBP was PnIA[R5, R9, L10, R14]) with the IC_50_ value of 1.2 nM. The most efficient competitor of [^125^I]-αBgt for binding to human α7 nAChR in GH_4_C_1_ cells was the analog PnIA[R9, L10] with IC_50_ = 270 nM. However, both of them showed an even higher affinity in electrophysiological experiments with IC_50_ values around 20 nM.

These data also confirm our previous results: Analysis of compounds with potential cholinergic activity in competition with radio-iodinated α-conotoxins is more convenient and allows obtaining more accurate values for the affinity of tested compounds, especially of those which have a relatively low affinity for nAChRs.

It is also interesting to compare the affinities measured either via competition with radio-iodinated α-Bgt or α-conotoxin PnIA analogs or by decrease in the nicotine-induced current. Radioligand analysis is the only choice when evaluating the affinity against AChBPs, but electrophysiology appears to be more preferable when working with the nAChRs because there is no underestimation of the affinity parameters.

### 3.3. nAChR -Inhibiting Peptides from Snake Venoms

**Figure 2:**
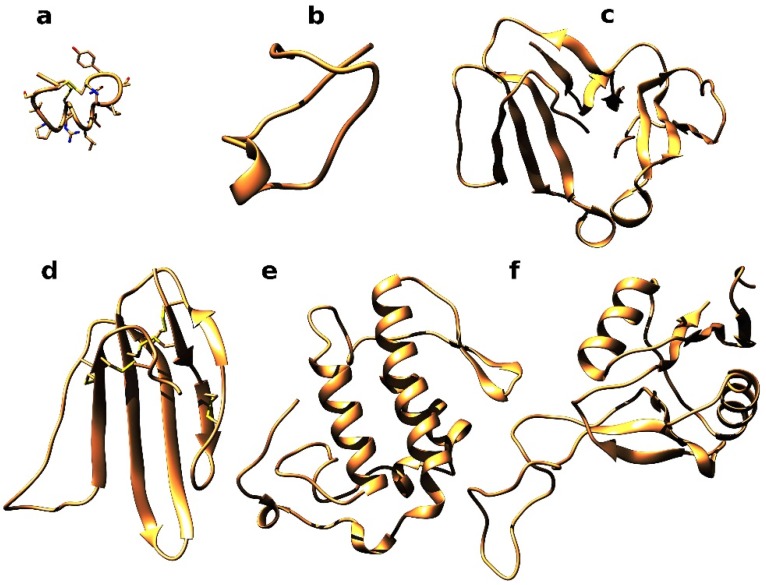
Experimental and modeled spatial structures of peptides and proteins of diverse structural classes interacting with nAChRs: **a**—molecular model of α-conotoxin PnIA[R9, L10] obtained with UCSF Chimera on the basis of PDB 1PEN, **b**—azemiopsin structure predicted by PEPFOLD service [65], **c**—dimeric α-cobratoxin X-ray structure [66], **d**—NMR structure of water-soluble Lynx1 domain [67], **e**—vurtoxin molecular model built by homology to ammodytoxin (PDB 3G8G), **f**—homology model for C-type lectin-like protein from *Bitis arietans* venom. Homology models are built using SWISS MODEL service [68].

It should be mentioned that some snake venoms also contain peptide neurotoxins, which can block nAChRs, although their number is much smaller than that of known α-conotoxins from *Conus* marine snails. Waglerins isolated from *Tropidolaemus wagleri pit-viper* [62] are composed of 22–24 amino acid residues with one disulfide bond. These peptides selectively block the adult (epsilon subunit-containing) form of the muscle nAChR. Recently, we characterized a peptide neurotoxin azemiopsin in the venom of *Azemiops feae* viper [63]. It consists of 21 residues, does not contain cysteine residues and shares a homologous C-terminal hexapeptide with waglerins (see Figure 2b for a putative structure representation). Azemiopsin efficiently and concentration-dependently blocked acetylcholine-induced currents in *Xenopus* oocytes heterologously expressing human muscle-type nAChR and was more potent against the adult form (α1β1εδ) than the fetal one (α1β1γδ) It competed with α-bungarotoxin for binding to both *Torpedo* and human α7 nAChR, being more active against the *Torpedo* receptor. Interestingly, two proline-rich peptides from the olive whip snake *Psammophis mossambicus*, post-translationally cleaved from the propeptide domain of a metalloproteinase precursor, manifested specific inhibition of mammalian neuronal α7 nAChR [64]. 

## 4. Proteins Interacting with nAChRs

### 4.1. Monomeric and Dimeric α-Neurotoxins and Non-Conventional Toxins

First of all two groups of the three-finger toxins from snake venoms should be mentioned here, namely α-neurotoxins and those weak (or non-conventional) toxins which target nAChRs [69]. α-Neurotoxins from the three-finger toxin family are classical ligands of nAChRs. Several recent reviews consider in details different properties of α-neurotoxins (see, for example, [70,71]). However, some new structural and functional variations among α-neurotoxins should be mentioned here. Thus, an atypical long-chain three-finger toxin, α-elapitoxin-Dpp2d, was isolated from the black mamba (*Dendroaspis polylepis*) venom [72]. This toxin has an amidated C-terminal arginine and potently inhibits α7 neuronal nAChR (IC_50_ 58 ± 24 nM) and muscle-type nAChR (IC_50_ 114 ± 37 nM), but does not affect α3β2 and α3β4 nAChRs at 1 μM concentrations. The activity data suggest that amidation does not significantly affect toxin selectivity. Two long chain three-finger toxins, α-elapitoxin-Aa2a and α-elapitoxin-Al2a were isolated from Australian elapids *Acanthophis antarcticus* and *Austrelaps labialis*, respectively [73,74]. Possessing a significant homology to the classical long-chain α-neurotoxins, α-elapitoxin-Al2a has only weak affinity for neuronal α7 nAChR (IC_50_ 1.2 μM) while α-elapitoxin-Aa2a practically lacks the affinity for this receptor (K_I_ 214 μM). 

Although the species difference for the interaction of α-neurotoxins with nAChRs was observed quite a long time ago [75], similar data for other neurotoxin types have only appeared recently. Thus, a non-conventional toxin denmotoxin has a much stronger specificity to chicken muscle nAChR than to mouse muscle nAChR [76]. Another example is fulgimotoxin from the venom of Green Vinesnake (*Oxybelis fulgidus*), which retains the canonical five disulfides of the non-conventional three-finger toxins and is highly neurotoxic to lizards; however, mice are unaffected [77]. 

Among relevantly new results is the discovery of covalently bound dimeric α-cobratoxin [78] (Figure 2c) and irditoxin, a covalently linked heterodimeric three-finger toxin [79]. The latter possesses taxon-specific lethality toward birds and lizards and was nontoxic toward mice. 

Historically, α-bungarotoxin derivatives have been used for histochemical staining of α7 nAChRs in the brain. However, the discovery of the high affinity of α-bungarotoxin to α9 and α9/α10 nAChRs requires an additional examination of the α-bungarotoxin histochemical staining specificity. A sound approach would be at first to probe the histochemical protocol using knockout tissues and then to proceed with its application for specific tasks, such as staining of α7 nAChRs on primary afferent neurons or on immature granule cells of the postnatal dentate gyrus. 

What should be emphasized is that the data showed that α-neurotoxins, considered to be specific labels for several nAChR subtypes, might also have some additional targets. For example, about a decade ago it was shown that α-bungarotoxin could bind to and inhibit one rare nonfunctional form of GABA_A_ receptor [80], but recently, at higher toxin concentrations, the inhibitory effects of α-bungarotoxin were also registered against the functional GABA_A_ subtypes [81]. 

### 4.2. Three-Finger Ly6 Proteins of the Non-Venomous Origin

There is another group of 3-finger proteins that interact with nAChRs. These are certain members of the large Ly6 family, such as Lynx1 (Figure 2d), SLURPs 1 and 2, Drosophila protein SLEEPLESS (SSS) and some others (see reviews [69,82,83,84]). Most of Ly6 proteins (Lynx1 among them) are attached to the membrane surface by a glycosylphosphatidylinositol (GPI) anchor, while some others (*i.e.*, SLURPs) are secreted water-soluble proteins. The mechanisms of Ly6–nAChR interactions are not yet clear: in most cases the result is a decreased activity associated with the nAChR involvement. There are theories that these modulatory actions are realized via binding of the Ly6 proteins to the ligand-binding domains of nAChRs, but the information about the binding modes is just starting to become clear [67,85]. On the other hand, there are recent data indicating that a modulation might proceed in the endoplasmic reticulum via the interactions with the nAChR subunits not yet assembled into the functional receptor complexes [86]. In a similar way, Ly6h protein in the rat brain influences functioning of α7 nAChR by diminishing its trafficking [87]. A common structural feature of all Ly6 proteins is the presence of an additional fifth disulfide not in the central loop II, like in the long-chain α-neurotoxins, but in the N-terminal loop I, like in the non-conventional neurotoxins [88]. In parallel to the earlier mentioned interactions of α-bungarotoxin not only with the α7 and muscle-type nAChRs but also with the GABA_A_ receptors, the Ly6 proteins also bind to other targets. A water-soluble analog of Lynx1, lacking its GPI anchor, interacts not only with the muscle-type and neuronal nAChRs, but also exerts a modulatory effect on the M3 muscarinic acetylcholine receptor [67]. Similarly, allosteric effects on the muscarinic acetylcholine receptors were registered for the non-conventional weak toxin WTX from the Naja kaouthia cobra venom [89], which is an efficient blocker of the muscle-type and neuronal α7 nAChRs [90]. Surprisingly, a sleep-inducing activity of the Drosophila Ly6 protein SLEEPLESS (SSS) is realized by attacking two absolutely different targets: Drosophila nAChRs (inhibition) and/or potassium Shaker channel (activation). *In vitro* experiments on the cell cultures have demonstrated that Lynx1 can also bind to these targets [91].

### 4.3. Snake Venom Phospholipases A_2_ and C-Type Lectin-like Proteins as Inhibitors of nAChRs

nAChRs are large molecules built of five membrane-spanning subunits. They have binding sites for different classes of compounds (agonists and competitive antagonists, positive and negative allosteric modulators, steroids and channel blockers) scattered over their exposed extracellular or cytoplasmic regions or hidden within the membrane. The variety of binding sites for low-molecular weight compounds does not seem surprising, but, as we have seen, large molecules like α-neurotoxins or Ly6 proteins can find the attachment sites on the nAChRs. Interestingly, even much larger molecules can bind to the nAChRs: We have recently shown that phospholipases A_2_ from the venoms of snakes belonging to different genera and families interact with the nAChRs of α7 and muscle types. First, it was shown that a protein, bitanarin, isolated from the venom of the puff adder *Bitis arietans* and possessing high phospholipolytic activity, blocked nAChRs of the α7 and muscle types [92]. Further studies of phospholipases A2 (Figure 2e) were performed with enzymes from the venoms of vipers *Vipera ursinii* and *V. nikolskii*, cobra *Naja kaouthia*, and krait *Bungarus fasciatus*. These studies demonstrated that all tested phospholipases A_2_ suppressed the acetylcholine- or cytisine-elicited currents in the *L. stagnalis* neurons and competed with α-bungarotoxin for binding to muscle- and neuronal α7-types of nAChR [93]. 

A continued exploration of compounds from Viperidae snake venoms that might interact with nAChRs resulted in identification of one more type of proteins capable of blocking nAChRs. These are the so-called C-type lectin-like proteins (CTLs, Figure 2f). We have shown that CTLs from the venoms of *B. arietans* and *Echis multisquamatus* block acetylcholine-induced currents in identified neurons of *L. stagnalis* [94]. Taking into account the above-mentioned ability of phospholipases A_2_ to block nAChRs, we can conclude that the snake venoms contain a broad range of proteins with different structures and activities capable of interacting with nAChRs with different affinities.

## 5. Conclusions

The results presented convincingly demonstrate that low-molecular weight compounds as well as peptide and protein neurotoxins are invaluable assistants to elucidate the mechanisms of nAChR functioning, to understand the reasons why their normal functions are disturbed and to try to find reliable ways to diagnostics and drug design. It was mentioned that it is the large size of nAChRs, built of five transmembrane subunits and having large ligand-binding and cytoplasmic domains, which creates a possibility of forming diverse binding sites with high or low affinity recognized by structurally very different compounds. In this mini-review we did not deal with the compounds which have binding sites within the membrane (channel blockers [95,96], some positive allosteric modulators [97,98], binding sites of local anesthetics [99,100]) or at the cytoplasmic surface (like rapsyn [101] or muscle-specific tyrosine kinase [102,103]), but concentrated on those which are bound at the nAChR *N*-terminal ligand-binding domain and act mostly as agonists or antagonists. The ideas of the three-dimensional structures of these sites in the different nAChR subtypes were formulated based mainly on the cryo-electron microscopic structure of the *Torpedo californica* nAChR [7], X-ray structures of the AChBP complexes and computer modeling [104], as well as on the X-ray structures of prokaryotic ligand-gated ion channels [105,106,107]. The correctness of this approach was recently confirmed by the X-ray structures of the Glu-Cl channel from *C. elegans* [108], as well as mammalian 5-HT3 [109] and GABAA [110] receptors with certain compounds at the ligand-binding sites. All these structures demonstrate that the ligand-binding site has a limited size, with the major role played by several aromatic residues (“aromatic box”). The available X-ray structures demonstrated that low-molecular-weight compounds, like nicotine, are practically completely buried in the binding site, whereas for larger molecules, such as peptide α-conotoxins, only about half of the molecule lies within the receptor. Similarly, a crucial contribution to the binding of protein α-neurotoxins comes from the tip of the central loop being immersed in the ligand-binding site. Thus, the discussion in this review of the low-molecular weight compounds together with peptides and proteins, all interacting with the ligand-binding sites, is justified because a lot of work is still required to identify the crucial common elements which would provide a higher affinity and better selectivity for the particular nAChR of interest in order to help in diagnosing and treating neurodegenerative and other diseases.

## References

[1] Changeux J.P. (2012). The nicotinic acetylcholine receptor: The founding father of the pentameric ligand-gated ion channel superfamily. J. Biol. Chem..

[2] Yakel J.L. (2013). Cholinergic receptors: Functional role of nicotinic ACh receptors in brain circuits and disease. Pflugers. Arch. Eur. J. Physiol..

[3] Corringer P.J., Poitevin F., Prevost M.S., Sauguet L., Delarue M., Changeux J.P. (2012). Structure and pharmacology of pentameric receptor channels: From bacteria to brain. Structure.

[4] Dineley K.T., Pandya A.A., Yakel J.L. (2015). Nicotinic ACh receptors as therapeutic targets in CNS disorders. Trends Pharmacol. Sci..

[5] Lombardo S., Maskos U. (2014). Role of the nicotinic acetylcholine receptor in Alzheimer’s disease pathology and treatment. Neuropharmacology.

[6] Farrugia M.E., Vincent A. (2010). Autoimmune mediated neuromuscular junction defects. Curr. Opin. Neurol..

[7] Unwin N. (2005). Refined structure of the nicotinic acetylcholine receptor at 4A resolution. J. Mol. Biol..

[8] Unwin N. (2013). Nicotinic acetylcholine receptor and the structural basis of neuromuscular transmission: Insights from Torpedo postsynaptic membranes. Q. Rev. Biophys..

[9] Brejc K., van Dijk W.J., Klaassen R.V., Schuurmans M., van Der Oost J., Smit A.B., Sixma T.K. (2001). Crystal structure of an ACh-binding protein reveals the ligand-binding domain of nicotinic receptors. Nature.

[10] Celie P.H.N., Kasheverov I.E., Mordvintsev D.Y., Hogg R.C., van Nierop P., van Elk R., van Rossum-Fikkert S.E., Zhmak M.N., Bertrand D., Tsetlin V. (2005). Crystal structure of nicotinic acetylcholine receptor homolog AChBP in complex with an alpha-conotoxin PnIA variant. Nat. Struct. Mol. Biol..

[11] Celie P.H.N., van Rossum-Fikkert S.E., van Dijk W.J., Brejc K., Smit A.B., Sixma T.K. (2004). Nicotine and carbamylcholine binding to nicotinic acetylcholine receptors as studied in AChBP crystal structures. Neuron.

[12] Hansen S.B., Sulzenbacher G., Huxford T., Marchot P., Taylor P., Bourne Y. (2005). Structures of Aplysia AChBP complexes with nicotinic agonists and antagonists reveal distinctive binding interfaces and conformations. EMBO J..

[13] Li S.-X., Huang S., Bren N., Noridomi K., Dellisanti C.D., Sine S.M., Chen L. (2011). Ligand-binding domain of an α7-nicotinic receptor chimera and its complex with agonist. Nat. Neurosci..

[14] Brams M., Pandya A., Kuzmin D., van Elk R., Krijnen L., Yakel J.L., Tsetlin V., Smit A.B., Ulens C. (2011). A structural and mutagenic blueprint for molecular recognition of strychnine and d-tubocurarine by different cys-loop receptors. PLoS Biol..

[15] Bourne Y., Talley T.T., Hansen S.B., Taylor P., Marchot P. (2005). Crystal structure of a Cbtx-AChBP complex reveals essential interactions between snake alpha-neurotoxins and nicotinic receptors. EMBO J..

[16] Huang S., Li S.-X., Bren N., Cheng K., Gomoto R., Chen L., Sine S.M. (2013). Complex between α-bungarotoxin and an α7 nicotinic receptor ligand-binding domain chimaera. Biochem. J..

[17] Ulens C., Hogg R.C., Celie P.H., Bertrand D., Tsetlin V., Smit A.B., Sixma T.K. (2006). Structural determinants of selective alpha-conotoxin binding to a nicotinic acetylcholine receptor homolog AChBP. Proc. Natl. Acad. Sci. USA.

[18] Dutertre S., Ulens C., Büttner R., Fish A., van Elk R., Kendel Y., Hopping G., Alewood P.F., Schroeder C., Nicke A. (2007). AChBP-targeted alpha-conotoxin correlates distinct binding orientations with nAChR subtype selectivity. EMBO J..

[19] Tsetlin V., Utkin Y., Kasheverov I. (2009). Polypeptide and peptide toxins, magnifying lenses for binding sites in nicotinic acetylcholine receptors. Biochem. Pharmacol..

[20] Utkin Y.N. (2013). Three-finger toxins, a deadly weapon of elapid venom--milestones of discovery. Toxicon.

[21] Shelukhina I.V., Kryukova E.V., Lips K.S., Tsetlin V.I., Kummer W. (2009). Presence of alpha7 nicotinic acetylcholine receptors on dorsal root ganglion neurons proved using knockout mice and selective alpha-neurotoxins in histochemistry. J. Neurochem..

[22] Frahm S., Slimak M.A., Ferrarese L., Santos-Torres J., Antolin-Fontes B., Auer S., Filkin S., Pons S., Fontaine J.-F., Tsetlin V. (2011). Aversion to nicotine is regulated by the balanced activity of β4 and α5 nicotinic receptor subunits in the medial habenula. Neuron.

[23] Olivera B.M. (2006). Conus peptides: Biodiversity-Based discovery and exogenomics. J. Biol. Chem..

[24] Kasheverov I.E., Utkin Y.N., Tsetlin V.I. (2009). Naturally occurring and synthetic peptides acting on nicotinic acetylcholine receptors. Curr. Pharm. Des..

[25] Prashanth J.R., Lewis R.J., Dutertre S. (2012). Towards an integrated venomics approach for accelerated conopeptide discovery. Toxicon.

[26] Otero A., Chapela M.-J., Atanassova M., Vieites J.M., Cabado A.G. (2011). Cyclic Imines: Chemistry and Mechanism of Action: A Review. Chem. Res. Toxicol..

[27] Bourne Y., Radic Z., Aráoz R., Talley T.T., Benoit E., Servent D., Taylor P., Molgó J., Marchot P. (2010). Structural determinants in phycotoxins and AChBP conferring high affinity binding and nicotinic AChR antagonism. Proc. Natl. Acad. Sci. USA.

[28] Wandscheer C.B., Vilariño N., Espiña B., Louzao M.C., Botana L.M. (2010). Human muscarinic acetylcholine receptors are a target of the marine toxin 13-desmethyl C spirolide. Chem. Res. Toxicol..

[29] Munday R., Quilliam M.A., LeBlanc P., Lewis N., Gallant P., Sperker S.A., Ewart H.S., MacKinnon S.L. (2012). Investigations into the toxicology of spirolides, a group of marine phycotoxins. Toxins (Basel).

[30] Kudryavtsev D., Makarieva T., Utkina N., Santalova E., Kryukova E., Methfessel C., Tsetlin V., Stonik V., Kasheverov I. (2014). Marine natural products acting on the acetylcholine-binding protein and nicotinic receptors: From computer modeling to binding studies and electrophysiology. Mar. Drugs.

[31] Molgó J., Aráoz R., Benoit E., Iorga B.I. (2013). Physical and virtual screening methods for marine toxins and drug discovery targeting nicotinic acetylcholine receptors. Expert Opin. Drug Discov..

[32] Philpot R.M. (2015). Potential Use of Nicotinic Receptor Agonists for the Treatment of Chemotherapy-Induced Cognitive Deficits. Neurochem. Res..

[33] Barbier A.J., Hilhorst M., van Vliet A., Snyder P., Palfreyman M.G., Gawryl M., Dgetluck N., Massaro M., Tiessen R., Timmerman W. (2015). Pharmacodynamics, Pharmacokinetics, Safety, and Tolerability of Encenicline, a Selective α7 Nicotinic Receptor Partial Agonist, in Single Ascending-dose and Bioavailability Studies. Clin. Ther..

[34] Dutta S., Hosmane B.S., Awni W.M. (2012). Population analyses of efficacy and safety of ABT-594 in subjects with diabetic peripheral neuropathic pain. AAPS J..

[35] Kasheverov I., Shelukhina I., Kudryavtsev D., Makarieva T., Spirova E., Guzii A., Stonik V., Tsetlin V. (2015). 6-Bromohypaphorine from Marine Nudibranch Mollusk Hermissenda crassicornis is an Agonist of Human α7 Nicotinic Acetylcholine Receptor. Mar. Drugs.

[36] Muttenthaler M., Akondi K.B., Alewood P.F. (2011). Structure-activity studies on alpha-conotoxins. Curr. Pharm. Des..

[37] Lebbe E.K.M., Peigneur S., Wijesekara I., Tytgat J. (2014). Conotoxins targeting nicotinic acetylcholine receptors: An overview. Mar. Drugs.

[38] Prashanth J.R., Brust A., Jin A.-H., Alewood P.F., Dutertre S., Lewis R.J. (2014). Cone snail venomics: from novel biology to novel therapeutics. Future Med. Chem..

[39] Del Bufalo A., Cesario A., Salinaro G., Fini M., Russo P. (2014). Alpha9 alpha10 nicotinic acetylcholine receptors as target for the treatment of chronic pain. Curr. Pharm. Des..

[40] Kauferstein S., Porth C., Kendel Y., Wunder C., Nicke A., Kordis D., Favreau P., Koua D., Stöcklin R., Mebs D. (2011). Venomic study on cone snails (Conus spp.) from South Africa. Toxicon.

[41] Peigneur S., van Der Haegen A., Möller C., Waelkens E., Diego-García E., Marí F., Naudé R., Tytgat J. (2013). Unraveling the peptidome of the South African cone snails Conus pictus and Conus natalis. Peptides.

[42] Peng C., Ye M., Wang Y., Shao X., Yuan D., Liu J., Hawrot E., Wang C., Chi C. (2010). A new subfamily of conotoxins belonging to the A-superfamily. Peptides.

[43] Luo S., Zhangsun D., Wu Y., Zhu X., Hu Y., McIntyre M., Christensen S., Akcan M., Craik D.J., McIntosh J.M. (2013). Characterization of a novel α-conotoxin from conus textile that selectively targets α6/α3β2β3 nicotinic acetylcholine receptors. J. Biol. Chem..

[44] Liu Z., Li H., Liu N., Wu C., Jiang J., Yue J., Jing Y., Dai Q. (2012). Diversity and evolution of conotoxins in Conus virgo, Conus eburneus, Conus imperialis and Conus marmoreus from the South China Sea. Toxicon.

[45] Inserra M.C., Kompella S.N., Vetter I., Brust A., Daly N.L., Cuny H., Craik D.J., Alewood P.F., Adams D.J., Lewis R.J. (2013). Isolation and characterization of α-conotoxin LsIA with potent activity at nicotinic acetylcholine receptors. Biochem. Pharmacol..

[46] Lebbe E.K.M., Peigneur S., Maiti M., Mille B.G., Devi P., Ravichandran S., Lescrinier E., Waelkens E., D’Souza L., Herdewijn P. (2014). Discovery of a new subclass of α-conotoxins in the venom of Conus australis. Toxicon.

[47] Nguyen B., le Caer J.-P., Aráoz R., Thai R., Lamthanh H., Benoit E., Molgó J. (2014). Isolation, purification and functional characterization of alpha-BnIA from Conus bandanus venom. Toxicon.

[48] Morales-González D., Flores-Martínez E., Zamora-Bustillos R., Rivera-Reyes R., Michel-Morfín J.E., Landa-Jaime V., Falcón A., Aguilar M.B. (2015). Diversity of A-conotoxins of three worm-hunting cone snails (Conus brunneus, Conus nux, and Conus princeps) from the Mexican Pacific coast. Peptides.

[49] Whiteaker P., Christensen S., Yoshikami D., Dowell C., Watkins M., Gulyas J., Rivier J., Olivera B.M., McIntosh J.M. (2007). Discovery, synthesis, and structure activity of a highly selective alpha7 nicotinic acetylcholine receptor antagonist. Biochemistry.

[50] Armishaw C.J., Singh N., Medina-Franco J.L., Clark R.J., Scott K.C.M., Houghten R.A., Jensen A.A. (2010). A synthetic combinatorial strategy for developing alpha-conotoxin analogs as potent alpha7 nicotinic acetylcholine receptor antagonists. J. Biol. Chem..

[51] Chang Y.-P., Banerjee J., Dowell C., Wu J., Gyanda R., Houghten R.A., Toll L., McIntosh J.M., Armishaw C.J. (2014). Discovery of a potent and selective α3β4 nicotinic acetylcholine receptor antagonist from an α-conotoxin synthetic combinatorial library. J. Med. Chem..

[52] Azam L., Yoshikami D., McIntosh J.M. (2008). Amino acid residues that confer high selectivity of the alpha6 nicotinic acetylcholine receptor subunit to alpha-conotoxin MII[S4A,E11A,L15A]. J. Biol. Chem..

[53] Hone A.J., Scadden M., Gajewiak J., Christensen S., Lindstrom J., McIntosh J.M. (2012). α-Conotoxin PeIA[S9H,V10A,E14N] potently and selectively blocks α6β2β3 versus α6β4 nicotinic acetylcholine receptors. Mol. Pharmacol..

[54] Olivera B.M., Quik M., Vincler M., McIntosh J.M. (2008). Subtype-selective conopeptides targeted to nicotinic receptors: Concerted discovery and biomedical applications. Channels (Austin).

[55] Yang K., Jin G., Wu J. (2009). Mysterious alpha6-containing nAChRs: Function, pharmacology, and pathophysiology. Acta Pharmacol. Sin..

[56] Pérez-Alvarez A., Hernández-Vivanco A., McIntosh J.M., Albillos A. (2012). Native α6β4* nicotinic receptors control exocytosis in human chromaffin cells of the adrenal gland. FASEB J..

[57] Surin A.M., Kriukova E.V, Strukov A.S., Zhmak M.N., Talka R., Tuominen R., Salminen O., Khiroug L., Kasheverov I.E., Tsetlin V.I. (2012). Effect of alpha-conotoxin MII and its *N*-terminal derivatives on Ca^2+^ and Na^+^ signals induced by nicotine in neuroblastoma cell line SH-SY5Y. Bioorg. Khim..

[58] Hone A.J., Whiteaker P., Mohn J.L., Jacob M.H., McIntosh J.M. (2010). Alexa Fluor 546-ArIB[V11L;V16A] is a potent ligand for selectively labeling alpha 7 nicotinic acetylcholine receptors. J. Neurochem..

[59] Kasheverov I.E., Zhmak M.N., Khruschov A.Y., Tsetlin V.I. (2011). Design of new α-conotoxins: From computer modeling to synthesis of potent cholinergic compounds. Mar. Drugs.

[60] Koromyslova A.D., Chugunov A.O., Efremov R.G. (2014). Deciphering fine molecular details of proteins’ structure and function with a Protein Surface Topography (PST) method. J. Chem. Inf. Model..

[61] Kasheverov I.E., Kudryavtsev D.S., Ivanov I.A., Zhmak M.N., Chugunov A.O., Tabakmakher V.M., Zelepuga E.A., Efremov R.G., Tsetlin V.I. (2015). Rational design of new ligands for nicotinic receptors on the basis of α-conotoxin PnIA. Dokl. Biochem. Biophys..

[62] Weinstein S.A., Schmidt J.J., Bernheimer A.W., Smith L.A. (1991). Characterization and amino acid sequences of two lethal peptides isolated from venom of Wagler’s pit viper, Trimeresurus wagleri. Toxicon.

[63] Utkin Y.N., Weise C., Kasheverov I.E., Andreeva T.V., Kryukova E.V., Zhmak M.N., Starkov V.G., Hoang N.A., Bertrand D., Ramerstorfer J. (2012). Azemiopsin from Azemiops feae viper venom, a novel polypeptide ligand of nicotinic acetylcholine receptor. J. Biol. Chem..

[64] Brust A., Sunagar K., Undheim E.A., Vetter I., Yang D.C., Yang D.C., Casewell N.R., Jackson T.N.W., Koludarov I., Alewood P.F. (2013). Differential evolution and neofunctionalization of snake venom metalloprotease domains. Mol. Cell. Proteomics.

[65] Thévenet P., Shen Y., Maupetit J., Guyon F., Derreumaux P., Tufféry P. (2012). PEP-FOLD: An updated de novo structure prediction server for both linear and disulfide bonded cyclic peptides. Nucleic Acids Res..

[66] Osipov A.V, Rucktooa P., Kasheverov I.E., Filkin S.Y., Starkov V.G., Andreeva T.V, Sixma T.K., Bertrand D., Utkin Y.N., Tsetlin V.I. (2012). Dimeric α-cobratoxin X-ray structure: Localization of intermolecular disulfides and possible mode of binding to nicotinic acetylcholine receptors. J. Biol. Chem..

[67] Lyukmanova E.N., Shenkarev Z.O., Shulepko M.A., Mineev K.S., D’Hoedt D., Kasheverov I.E., Filkin S.Y., Krivolapova A.P., Janickova H., Dolezal V. (2011). NMR structure and action on nicotinic acetylcholine receptors of water-soluble domain of human LYNX1. J. Biol. Chem..

[68] Guex N., Peitsch M.C., Schwede T. (2009). Automated comparative protein structure modeling with SWISS-MODEL and Swiss-PdbViewer: A historical perspective. Electrophoresis.

[69] Tsetlin V.I. (2015). Three-finger snake neurotoxins and Ly6 proteins targeting nicotinic acetylcholine receptors: Pharmacological tools and endogenous modulators. Trends Pharmacol. Sci..

[70] Kini R.M., Doley R. (2010). Structure, function and evolution of three-finger toxins: Mini proteins with multiple targets. Toxicon.

[71] Barber C.M., Isbister G.K., Hodgson W.C. (2013). Alpha neurotoxins. Toxicon.

[72] Wang C.I., Reeks T., Vetter I., Vergara I., Kovtun O., Lewis R.J., Alewood P.F., Durek T. (2014). Isolation and Structural and Pharmacological Characterization of α-Elapitoxin-Dpp2d, an Amidated Three Finger Toxin from Black Mamba Venom. Biochemistry.

[73] Blacklow B., Kornhauser R., Hains P.G., Loiacono R., Escoubas P., Graudins A., Nicholson G.M. (2011). α-Elapitoxin-Aa2a, a long-chain snake α-neurotoxin with potent actions on muscle (α1)(2)βγδ nicotinic receptors, lacks the classical high affinity for neuronal α7 nicotinic receptors. Biochem. Pharmacol..

[74] Marcon F., Leblanc M., Vetter I., Lewis R.J., Escoubas P., Nicholson G.M. (2012). Pharmacological characterization of α-elapitoxin-Al2a from the venom of the Australian pygmy copperhead (*Austrelaps. labialis*): An atypical long-chain α-neurotoxin with only weak affinity for α7 nicotinic receptors. Biochem. Pharmacol..

[75] Ishikawa Y., Kano M., Tamiya N., Shimada Y. (1985). Acetylcholine receptors of human skeletal muscle: A species difference detected by snake neurotoxins. Brain Res..

[76] Pawlak J., Mackessy S.P., Fry B.G., Bhatia M., Mourier G., Fruchart-Gaillard C., Servent D., Ménez R., Stura E., Ménez A. (2006). Denmotoxin, a three-finger toxin from the colubrid snake Boiga dendrophila (Mangrove Catsnake) with bird-specific activity. J. Biol. Chem..

[77] Heyborne W.H., Mackessy S.P. (2013). Identification and characterization of a taxon-specific three-finger toxin from the venom of the Green Vinesnake (Oxybelis fulgidus; Family Colubridae). Biochimie.

[78] Osipov A.V., Kasheverov I.E., Makarova Y.V., Starkov V.G., Vorontsova O.V., Ziganshin R.K., Andreeva T.V., Serebryakova M.V., Benoit A., Hogg R.C. (2008). Naturally occurring disulfide-bound dimers of three-fingered toxins: A paradigm for biological activity diversification. J. Biol. Chem..

[79] Pawlak J., Mackessy S.P., Sixberry N.M., Stura E.A., Le Du M.H., Menez R., Foo C.S., Menez A., Nirthanan S., Kini R.M. (2009). Irditoxin, a novel covalently linked heterodimeric three finger toxin with high taxon-specific neurotoxicity. FASEB J..

[80] McCann C.M., Bracamontes J., Steinbach J.H., Sanes J.R. (2006). The cholinergic antagonist alpha-bungarotoxin also binds and blocks a subset of GABA receptors. Proc. Natl. Acad. Sci. USA.

[81] Hannan S., Mortensen M., Smart T.G. (2015). Snake neurotoxin α-bungarotoxin is an antagonist at native GABA_A_ receptors. Neuropharmacology.

[82] Miwa J.M., Freedman R., Lester H.A. (2011). Neural systems governed by nicotinic acetylcholine receptors: Emerging hypotheses. Neuron.

[83] Ibañez-Tallon I., Nitabach M.N. (2012). Tethering toxins and peptide ligands for modulation of neuronal function. Curr. Opin. Neurobiol..

[84] Miwa J.M., Lester H.A., Walz A. (2012). Optimizing cholinergic tone through lynx modulators of nicotinic receptors: Implications for plasticity and nicotine addiction. Physiology (Bethesda).

[85] Lyukmanova E.N., Shulepko M.A., Buldakova S.L., Kasheverov I.E., Shenkarev Z.O., Reshetnikov R.V., Filkin S.Y., Kudryavtsev D.S., Ojomoko L.O., Kryukova E.V. (2013). Water-soluble LYNX1 residues important for interaction with muscle-type and/or neuronal nicotinic receptors. J. Biol. Chem..

[86] Nichols W.A., Henderson B.J., Yu C., Parker R.L., Richards C.I., Lester H.A., Miwa J.M. (2014). Lynx1 shifts α4β2 nicotinic receptor subunit stoichiometry by affecting assembly in the endoplasmic reticulum. J. Biol. Chem..

[87] Puddifoot C.A., Wu M., Sung R.-J., Joiner W.J. (2015). Ly6h regulates trafficking of alpha7 nicotinic acetylcholine receptors and nicotine-induced potentiation of glutamatergic signaling. J. Neurosci..

[88] Nirthanan S., Gopalakrishnakone P., Gwee M.C.E., Khoo H.E., Kini R.M. (2003). Non-conventional toxins from Elapid venoms. Toxicon.

[89] Mordvintsev D.Y., Polyak Y.L., Rodionov D.I., Jakubik J., Dolezal V., Karlsson E., Tsetlin V.I., Utkin Y.N. (2009). Weak toxin WTX from Naja kaouthia cobra venom interacts with both nicotinic and muscarinic acetylcholine receptors. FEBS J..

[90] Utkin Y.N., Kukhtina V.V, Kryukova E.V, Chiodini F., Bertrand D., Methfessel C., Tsetlin V.I. (2001). “Weak toxin” from Naja kaouthia is a nontoxic antagonist of alpha 7 and muscle-type nicotinic acetylcholine receptors. J. Biol. Chem..

[91] Wu M., Robinson J.E., Joiner W.J. (2014). SLEEPLESS is a bifunctional regulator of excitability and cholinergic synaptic transmission. Curr. Biol..

[92] Vulfius C.A., Gorbacheva E.V., Starkov V.G., Osipov A.V., Kasheverov I.E., Andreeva T.V., Astashev M.E., Tsetlin V.I., Utkin Y.N. (2011). An unusual phospholipase A2 from puff adder Bitis arietans venom—A novel blocker of nicotinic acetylcholine receptors. Toxicon.

[93] Vulfius C.A., Kasheverov I.E., Starkov V.G., Osipov A.V., Andreeva T.V., Filkin S.Y., Gorbacheva E.V., Astashev M.E., Tsetlin V.I., Utkin Y.N. (2014). Inhibition of nicotinic acetylcholine receptors, a novel facet in the pleiotropic activities of snake venom phospholipases A2. PLoS ONE.

[94] Vulfius C.A., Starkov V.G., Andreeva T.V., Tsetlin V.I., Utkin Yu.N. (2015). Novel antagonists of nicotinic acethylcholine receptors—Proteins from venoms of viperidae snakes. Dokl. Biochem. Biophys..

[95] Bixel M.G., Weise C., Bolognesi M.L., Rosini M., Brierly M.J., Mellor I.R., Usherwood P.N.R., Melchiorre C., Hucho F. (2000). Location of the polyamine binding site in the vestibule of the nicotinic acetylcholine receptor ion channel. J. Biol. Chem..

[96] Pandhare A., Hamouda A.K., Staggs B., Aggarwal S., Duddempudi P.K., Lever J.R., Lapinsky D.J., Jansen M., Cohen J.B., Blanton M.P. (2012). Bupropion binds to two sites in the Torpedo nicotinic acetylcholine receptor transmembrane domain: A photoaffinity labeling study with the bupropion analogue [(125)I]-SADU-3–72. Biochemistry.

[97] Collins T., Young G., Millar N. (2011). Competitive binding at a nicotinic receptor transmembrane site of two α7-selective positive allosteric modulators with differing effects on agonist-evoked. Neuropharmacology.

[98] Pandya A.A., Yakel J.L. (2013). Effects of neuronal nicotinic acetylcholine receptor allosteric modulators in animal behavior studies. Biochem. Pharmacol..

[99] Wang H., Zhang Y., Li S.-T. (2010). The effect of local anesthetics on the inhibition of adult muscle-type nicotinic acetylcholine receptors by nondepolarizing muscle relaxants. Eur. J. Pharmacol..

[100] Alberola-Die A., Reboreda A., Lamas J.A., Morales A. (2013). Lidocaine effects on acetylcholine-elicited currents from mouse superior cervical ganglion neurons. Neurosci. Res..

[101] Zuber B., Unwin N. (2013). Structure and superorganization of acetylcholine receptor-rapsyn complexes. Proc. Natl. Acad. Sci. USA.

[102] Punga A.R., Maj M., Lin S., Meinen S., Rüegg M.A. (2011). MuSK levels differ between adult skeletal muscles and influence postsynaptic plasticity. Eur. J. Neurosci..

[103] Stiegler A.L., Burden S.J., Hubbard S.R. (2009). Crystal structure of the frizzled-like cysteine-rich domain of the receptor tyrosine kinase MuSK. J. Mol. Biol..

[104] Akdemir A., Edink E., Thompson A.J., Lummis S.C.R., Kooistra A.J., de Graaf C., de Esch I.J.P. (2012). Identification of novel α7 nicotinic receptor ligands by in silico screening against the crystal structure of a chimeric α7 receptor ligand binding domain. Bioorg. Med. Chem..

[105] Hilf R.J.C., Dutzler R. (2008). X-ray structure of a prokaryotic pentameric ligand-gated ion channel. Nature.

[106] Bocquet N., Nury H., Baaden M., le Poupon C., Changeux J.-P., Delarue M., Corringer P.-J. (2009). X-ray structure of a pentameric ligand-gated ion channel in an apparently open conformation. Nature.

[107] Nury H., van Renterghem C., Weng Y., Tran A., Baaden M., Dufresne V., Changeux J.-P., Sonner J.M., Delarue M., Corringer P.-J. (2011). X-ray structures of general anaesthetics bound to a pentameric ligand-gated ion channel. Nature.

[108] Hibbs R.E., Gouaux E. (2011). Principles of activation and permeation in an anion-selective Cys-loop receptor. Nature.

[109] Hassaine G., Deluz C., Grasso L., Wyss R., Tol M.B., Hovius R., Graff A., Stahlberg H., Tomizaki T., Desmyter A. (2014). X-ray structure of the mouse serotonin 5-HT3 receptor. Nature.

[110] Miller P.S., Aricescu A.R. (2014). Crystal structure of a human GABAA receptor. Nature.

